# Distribution patterns, carbon sources and niche partitioning in cave shrimps (Atyidae: *Typhlatya*)

**DOI:** 10.1038/s41598-020-69562-2

**Published:** 2020-07-30

**Authors:** E. M. Chávez-Solís, C. Solís, N. Simões, M. Mascaró

**Affiliations:** 1 Posgrado en Ciencias Biológicas, Unidad de Posgrado, Edificio A, 1er piso, Circuito de Posgrados, Ciudad Universitaria, Coyoacán, C.P. 04510 Ciudad de México, Mexico; 2 Unidad Multidisciplinaria de Docencia e Investigación, Facultad de Ciencias, Universidad Nacional Autónoma de México, Puerto de Abrigo S/N, C.P. 97355 Sisal, Yucatán Mexico; 3 Laboratorio Nacional de Espectrometría de Masas con Aceleradores (LEMA), Instituto de Física, Circuito de la Investigación Científica, Universidad Nacional Autónoma de México, Ciudad Universitaria, C.P. 04510 Ciudad de México, México; 4 National Coastal Resilience Laboratory (LANRESC), Puerto de Abrigo S/N, C.P. 97355 Sisal, Yucatán Mexico; 5 International Chair for Ocean and Coastal Studies, Harte Research Institute, Texas A&M at Corpus Christi, Corpus Christi, TX USA

**Keywords:** Ecology, Zoology, Biogeochemistry, Environmental sciences

## Abstract

Cave shrimps of the *Typhlatya* genus are common and widespread in fresh, brackish and marine groundwater throughout the Yucatan Peninsula (Mexico). These species are ideal models to test niche partitioning within sympatric species in oligotrophic systems. Nevertheless, their food sources remain unidentified, and despite their frequency and functional importance, distribution and abundance patterns of these species within caves have not been fully recognized. Here, we describe the abundance of three *Typhlatya* species in different temporal and spatial scales, investigate changes in water conditions, and potential sources of carbon as an indication of food origin. Species composition and abundance varied markedly in space and time revealing patterns that differed from one system to another and in relation to environmental parameters. Isotope analysis showed that each species reflects a particular δ^13^C and Δ^14^C fingerprint, suggesting they feed in different proportions from the available carbon sources. Overall, our findings suggest a niche partitioning of habitat and feeding sources amongst the three *Typhlatya* species investigated, where environmental characteristics and physiological differences could play an important role governing their distribution patterns.

## Introduction

The lack of photosynthesis in caves and the resulting limitation in food sources is one of the strongest selection pressures and drivers of evolution for life in caves^1^. Competition for nutrients in oligotrophic environments, such as anchialine ecosystems—defined as subterranean estuaries that extend inland to the limit of seawater penetration^2^, certainly requires a unique set of specialization traits that allow for niche partitioning amongst stygobionts (aquatic species strictly bound to the subterranean habitat). A closer look at stygobiont biodiversity reveals that co-occurrence of sympatric species within the same system is rare but can be occasionally observed^3,4^. This work gathers evidence that suggests how three sympatric cave shrimp species that coexist in groundwater ecosystems have partitioned their niche to avoid competitive exclusion.

Groundwater in the anchialine systems of Yucatan is vertically stratified by the intrusion of marine water from the coast which inserts below the freshwater that constantly flows towards the coast^2^. This marked halocline, along with changes in temperature, dissolved oxygen, pH and redox potential has been considered a physicochemical barrier that separates freshwater and saline communities^5,6^. This stratification is expected in coastal systems, and as distance from the coast increases, the marine intrusion is found deeper and only a few deep cenotes inland may reach the saline layers^7^.

In terms of energy production and food webs, the sun-influenced *cenote pools*—cenote is a Mayan derived name for local sinkholes—are the only sites where photosynthesis can take place and allochthonous organic matter can enter the system. Therefore, these ecosystems have been historically regarded as dependent of allochthonous input. However, recent investigations have changed the oligotrophic perspective of the aphotic cave passages and recognize chemosynthesis by microbes in caves^8^, suggesting a vital role to the stygobitic trophic structure.

Stable isotope analysis has been used to track the origin, flow and transformations of energy through trophic webs^9–11^. We used the isotope fractionation principle to identify carbon sources in groundwater and relate them to the carbon composition in *Typhlatya* species collected from fresh and marine groundwater habitats. Stable carbon (δ^13^C) signatures from terrestrial and aquatic photosynthesis, however, may overlap and preclude the discrimination of carbon sources^12^.

Plants maintain a constant radiocarbon content as they incorporate carbon from the atmosphere. When they die, ^14^C begins to decay at a rate governed by a half-life of 5,730 years^13,14^. While “modern” radiocarbon content (atmospheric or recently incorporated through photosynthesis) in a sample is similar to atmospheric concentration, complex soil dynamics can hold chemically labile organic carbon that, despite its age (lower ^14^C content than modern carbon), may still be available for microbial catabolism^12^. Dissolved inorganic carbon (DIC)—carbon dioxide (CO_2_) and carbonates (CO_3_)—in karstic groundwater habitats is most likely to be derived by the weathering of the calcareous limestone, which is ^14^C depleted (considered “radiocarbon-dead carbon”)^12^. This leads to a contrasting radiocarbon content in autotrophs that incorporate DIC (i.e. algae and chemosynthesis by microbes) in contrast with plants incorporating atmospheric CO_2_, which will be reflected in their consumers. Therefore, the use of radiocarbon together with stable isotopes in food sources and consumers can shed light on the structure of food webs. Species feeding on different sources are likely to have contrasting stable isotopes or radiocarbon content, a condition that may help explain the ecological niche and distribution patterns of *Typhlatya* populations in groundwater ecosystems.

In this work we used the *Typhlatya* genus as a biological model to study distribution patterns within caves and feeding sources which are available in the groundwater. *Typhlatya* species are some of the most common and widespread stygobionts in the Yucatan Peninsula. They have been observed in fresh, brackish and marine groundwater, both in coastal and inland systems^3,15^. They are key in the energy transfer to higher trophic levels as they graze or filter feed on small particles or microbial mats^10^, and have been observed in cenote pools and aphotic cave passages where the absence of light precludes photosynthesis. They are sufficiently large to be conspicuous and may be identified underwater by the unaided eye. Notwithstanding their frequent occurrence and their trophic importance, patterns in their distribution within caves and factors associated have not been fully recognized.

The aims of this work were to describe the distribution and abundance patterns of *Typhlatya mitchelli, T. pearsei* and *T. dzilamensis* in two spatial scales (depth and geography of the Yucatan Peninsula) and two temporal scales (daily and seasonal). The purpose was to correlate these features with changes in physical and chemical water conditions, as well as potential sources of carbon as an indication of food origin. In addition, we aimed to test if these species had daily movements within sunlight influenced regions and whether meteorological seasons influenced their populations. Linking distribution patterns and feeding sources with environmental data will help understand the community structure, functional role and niche partitioning of ecologically similar sympatric species of this widespread *Typhlatya* genus in Yucatan.

## Results

**Figure 1:**
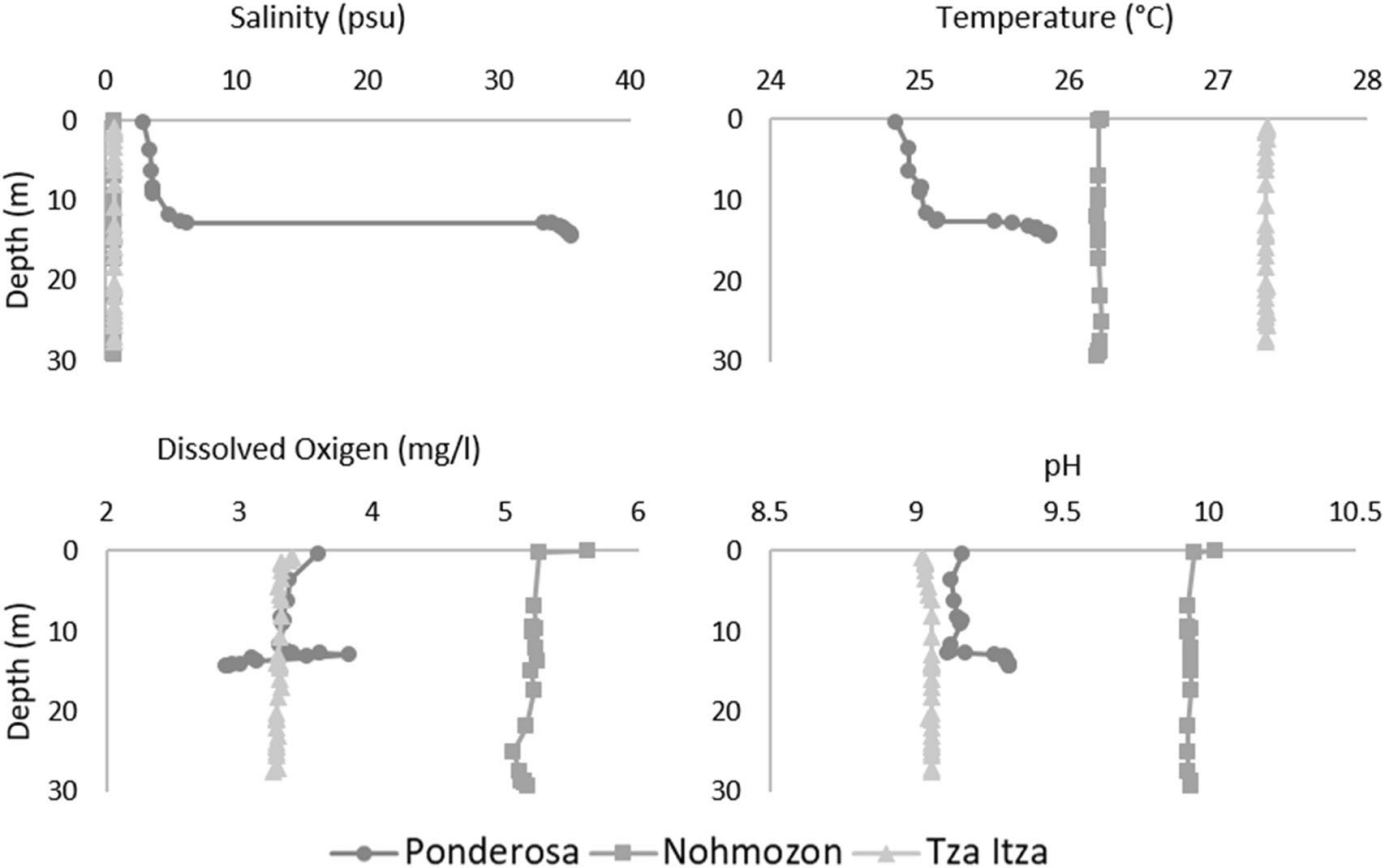
Vertical profile of physical and chemical hydrological parameters of Ponderosa Nohmozon and Tza Itza Systems. Data obtained using a Hydrolab DataSonde5.

Tza Itza, Nohmozon and Kankirixche are all freshwater systems, whereas Ponderosa is a vertically stratified anchialine system with the presence of a halocline (Fig. 1). Parameters in Tza Itza and Nohmozon were constant throughout the water column and differed among systems. Overall, temperature was highest in Tza Itza, followed by Nohmozon and finally, Ponderosa had the lowest thermal values. Dissolved oxygen was highest in Nohmozon and was similar in Ponderosa and Tza Itza. Observed pH was greatest in Nohmozon, with values close to 10, whilst Tza Itza and Ponderosa had values closer to 9. Ponderosa was vertically stratified in all parameters with a marked cline around 13 m of depth (Fig. 1). Salinity increased slowly from 3 psu at the surface to 6 psu at 13 m, and raised abruptly to 36 psu at 14 m, resulting in a marked halocline. Temperature was slightly under 25 °C at the surface and showed an abrupt increase of 1 °C across the halocline. Dissolved oxygen increased at the upper limit of the halocline from 3.4 to 4 mg/l and decreased in the saline layer to 2.8 mg/l. Finally, pH changed from the freshwater to the saline layer, by first dropping slightly then increasing from 9.2 to 9.4.

### Spatial distribution of *Typhlatya* species within anchialine systems

**Figure 2:**
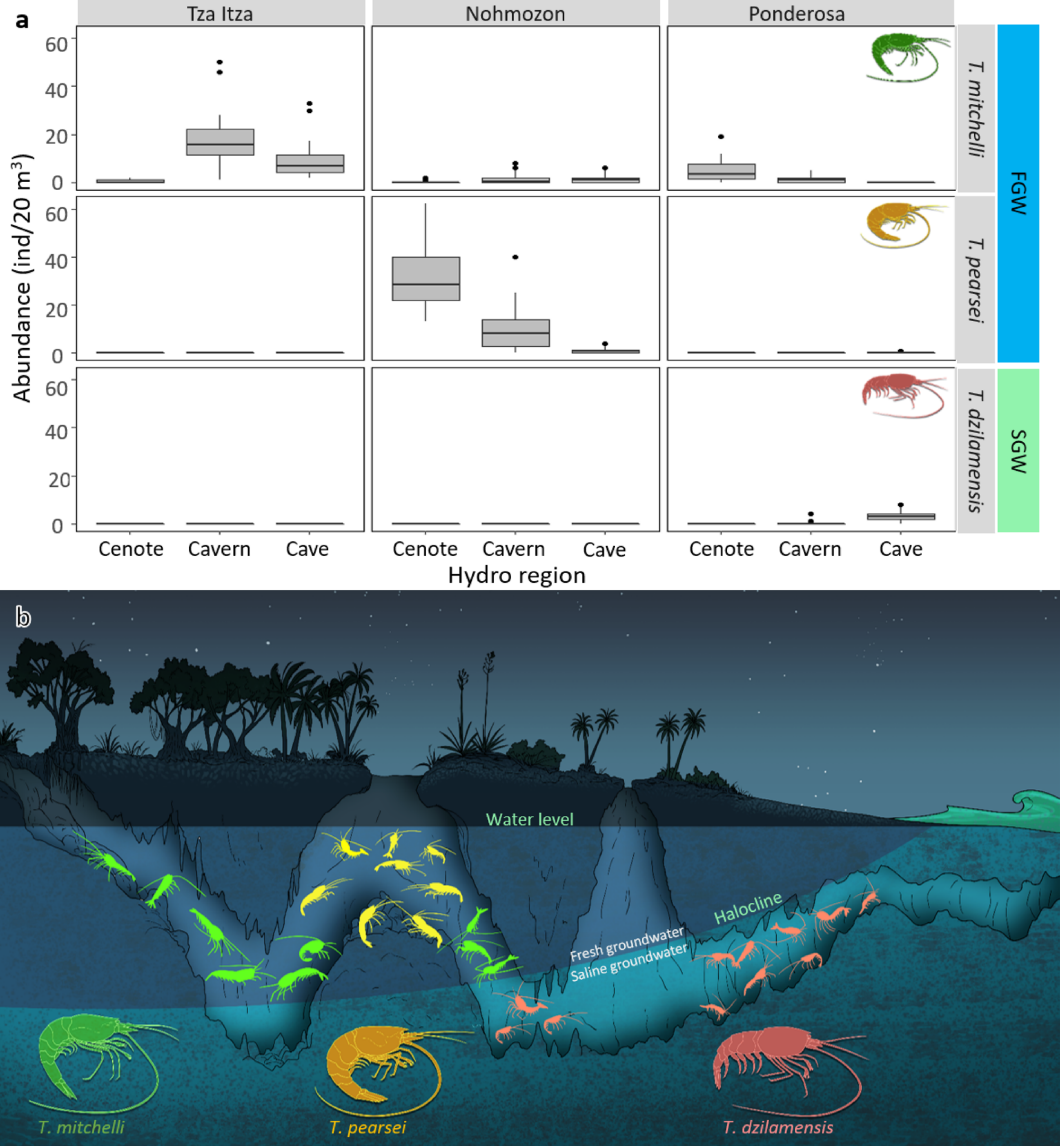
*Typhlatya* hydro region distribution in Yucatan underground submerged systems. (**A**) Amount of observed individuals per transect of 20 m^3^ at the cenote pool, cavern and cave hydro regions of Nohmozon, Tza Itza and Ponderosa systems. The boxplots indicate the first and third quartile of the data, the black line indicates the median and the whiskers extend to the most extreme data which is no further than 2 standard deviations. (**B**) Visualization of species distribution in a theoretical continuous underground system, showing the freshwater cenotes farther from the coast and the saline intrusion being shallower as closer to the coast. Artwork by Alberto Guerra.

The abundance and presence of *Typhlatya* species changed between hydro-regions in a way that differed from one system to another (Fig. 2A, B), as indicated by the significant interaction term in Table 1 (Sy × Hr). *Typhlatya mitchelli* was present in all three systems, but was most abundant in the cavern of Tza Itza. Here, its abundance decreased in the cave and was rarely found in the cenote pool. In Nohmozon and Ponderosa, *T. mitchelli* was consistently observed in relatively low abundance both in the cavern and in the cenote pool (Fig. 2A). This suggests *T. mitchelli* is mostly distributed in the cavern and cave as close to the cenote pool, but avoiding it (Fig. 2B). *Typhlatya pearsei* was only observed at night. This species was primarily found in Nohmozon, it occurred only once in Ponderosa and was never registered in Tza Itza. The highest abundance of all species registered in this study corresponded to *T. pearsei* in the cenote pool of Nohmozon, and decreased in numbers through the cavern and cave (Fig. 2A). *Typhlatya dzilamensis* was found mostly under the halocline in the cave of Ponderosa (Fig. 2B). It was only rarely observed in the cavern, and never observed in the cenote pool (Fig. 2A). *Typhlatya dzilamensis* was also the species with the lowest abundance of all three species in this study.

**Table 1:** Permutational Multivariate ANOVA (PERMANOVA) results on distribution data of *Typhlatya* species within underground systems of Yucatan.

Source	df	SS	MS	Pseudo-F	*p*	Unique permutations
System (Sy)	2	99,424	49,712	96.7	0.0001***	9,944
Hydro region (Hr)	2	21,191	10,596	20.6	0.0001***	9,944
Season (Se)	2	2,110	1,055	2.2	0.0822	9,959
Sy × Hr	4	89,856	22,464	43.8	0.0001***	9,933
Sy × Se	4	2,519	630	1.4	0.2359	9,947
Hr × Se	4	1858	464	1.0	0.4174	9,959
Sy × Hr × Se	8	1723	215	0.5	0.8665	9,946
Obs (Sy × Hr × Se)	38	15,310	403	1.7	0.0037***	9,857
Residuals	169	40,086	237			
Total	233	274,000				

Sources are: System (Sy), Hydro region (Hr), Season (Se) and Observer (Obs). Asterisks refer to the probability of that *p* value.

***p < 0.001.

Species composition did not vary markedly from one season to another (Table 1) and spatial differences in the abundance of all three *Typhlatya*, both between hydro-regions and systems were remarkably constant throughout the year (Se, Sy × Se, Hr × Se; Table 1). Because the random factor “observer” was nested within the combinations of all three fixed factors, results of the permutational multivariate ANOVA are fully interpretable (Obs; Table 1).

*Typhlatya* species composition was unique in each hydro-region at each system, as further confirmed by pair-wise t-test comparing hydro-regions within each system which showed consistent statistical differences (Table 2 and Fig. 2).

**Table 2:** Pair-wise t-tests for system and hydro region terms with each factor level.

	Cenote pool	Cavern
**Tza Itza**		
*Cavern*	15.1***	
*Cave*	13.8***	2.9*
**Nohmozon**		
*Cavern*	2.4**	
*Cave*	6.7***	2.6**
**Ponderosa**		
*Cavern*	3.0*	
*Cave*	10.5***	7.6***

t-values contrasting each hydro region within the same cenote are provided. Symbols refer to the probability of t-values.

*p < 0.05; **p < 0.01; ***p < 0.001.

### Carbon tracing in *Typhlatya* tissue

The Carbon (C) and Nitrogen (N) percent in tissue composition of all three *Typhlatya* species was similar and ranged from 48 to 63.6% and 3.6 to 12.4% for C and N, respectively (Supplementary Figure SF1). *Typhlatya mitchelli* showed a consistently lower C/N mean ratio of 4.7 (± 0.6) in comparison to *T. pearsei* and *T. dzilamensis*, which resulted in 9.8 (± 6.2) and 8.2 (± 2.4) respectively.

**Figure 3:**
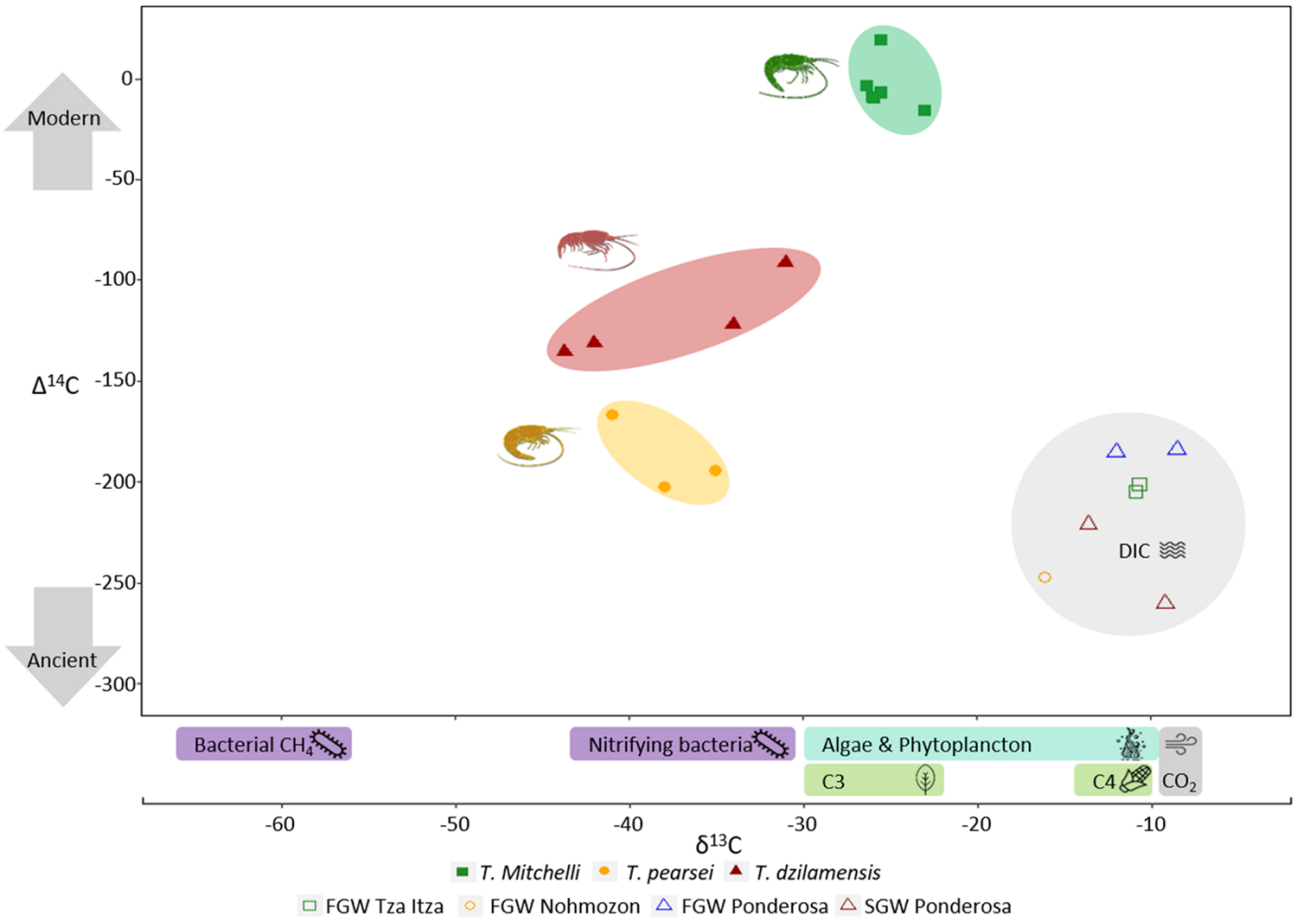
Carbon isotopic analysis indicating the Δ^14^C and δ^13^C bulk composition of *T. mitchelli* (sampled in Tza Itza), *T. pearsei* (sampled in Nohmozon) and *T. dzilamensis* (sampled in Ponderosa), and DIC contained in fresh groundwater (FGW) and saline groundwater (SGW), accordingly. δ^13^C values of potential carbon sources are indicated as ranges (source data from^8,11,16,17^). Artwork by Alberto Guerra.

A broad range of accelerator mass spectrometry (AMS)-δ^13^C and Δ^14^C values were obtained from the biomass of *Typhlatya* species. *Typhlatya mitchelli* from Tza Itza (n = 6) had δ^13^C values ranging from − 23.0 ± 2 to − 26.3 ± 1.4‰. *Typhlatya pearsei* from Nohmozon showed lower values ranging from δ^13^C − 35.0 ± 1 to − 41.0 ± 1‰ (n = 3) (Fig. 3). Similarly, *T. dzilamensis* collected from the saline groundwater layer in Ponderosa showed δ^13^C values that varied from − 31.0 ± 0.3 to − 44.0 ± 0.6‰ (n = 4) (Fig. 3). Values of δ^13^C obtained via AMS were always within the ranges obtained using IRMS δ^13^C, hence were used as indicators in the analysis of food sources in *Typhlatya* biomass contribution (Supplementary Table ST1).

The δ^13^C in dissolved inorganic carbon (DIC) in fresh groundwater (FGW) ranged from − 16.1‰ (± 0.8) in a sample taken at Nohmozon to an average − 10.8‰ (± 0.1) at Tza Itza and − 10.3‰ (± 2.5) at Ponderosa. δ^13^C in DIC in saline groundwater (SGW) taken from Ponderosa showed average value of − 11‰ (± 3.1) (Fig. 3).

*Typhlatya mitchelli* from Tza Itza showed Δ^14^C values ranging from − 3.5 to 19.2‰ (percentage of modern carbon (pMC) of 99.0 ± 0.3 to 103.0 ± 0.3%) corresponding to a modern age. *Typhlatya pearsei* collected in FGW at Nohmozon had Δ^14^C values from − 202 to − 166‰ (pMC of 80.4 ± 0.3% to 91.6 ± 0.3%), equivalent to an apparent age of − 1,750 to − 1,400 years before present (yBP). *Typhlatya dzilamensis* collected in SGW at Ponderosa showed Δ^14^C values from − 91.5 to − 135.5‰ (pMC of 91.6 ± 0.3% to 87.1 ± 0.3%), equivalent to a range of 700 to 1,100 yBP (Fig. 3). These results indicate there is a difference in Δ^14^C trace corresponding to each of the observed species. While *T. mitchelli* incorporates carbon from a modern source, *T. pearsei* and *T. dzilamensis* are partially incorporating carbon from an old source.

DIC in FGW of Tza Itza showed an average of Δ^14^C =  − 203‰ (pMC of 82.2 ± 0.3%), corresponding to an apparent age of 1759 yBP. DIC in FGW in the cenote pool of Nohmozon had a Δ^14^C of − 247‰ (pMC of 75.9 ± 0.3), corresponding to an apparent age of 2,213 yBP. DIC in FGW of Ponderosa had a Δ^14^C of − 185‰, corresponding to an apparent age of 1576 yBP, while SGW had Δ^14^C of − 260‰ (pMC of 74.7 ± 0.3), with an apparent age of 2,765 yBP (Fig. 3). Despite the fact that *T. mitchelli* inhabits groundwater that is ^14^C depleted in Tza Itza, it has a modern Δ^14^C trace, suggesting it is feeding on a modern source. *Typhlatya pearsei* and *T. dzilamensis* show older apparent ages, which are closer to the Δ^14^C of DIC of their specific habitat, suggesting that they are feeding on sources that can uptake DIC available in groundwater.

#### Mixing models

Biomass values of δ^13^C and ∆^14^C, as well as values published for potential sources were incorporated into our mixing model under two scenarios. Organic matter (OM) was established as δ^13^C_OM_ =  − 25.3‰ and Δ^14^C_OM_ =  − 4.3‰, obtained from the average values of *T. mitchelli* in Tza Itza, which represent the closest value to the photosynthetic and modern organic matter from terrestrial and aquatic sources measured in this study (Table 3). The oldest DIC measured in this study was from the saline groundwater of Ponderosa, thus, our second scenario was modelled with an ancient methane of Δ^14^C_AM2_ =  − 260‰ (Table 3).

**Table 3:** Two mixing model scenarios considering accelerator mass spectrometry (AMS) δ^13^C and Δ^14^C of modern organic matter (OM), modern methane (MM) and ancient methane (AM) as endmember potential sources that contribute to *Typhlatya* diet affecting their tissue composition.

	Scenario	1	2
	δ^13^C (‰)	Δ^14^C (‰)	Δ^14^C (‰)
OM	− 25.4	− 4.3	− 4.3
MM	− 66.3	0	0
AM	− 56.3	− 1,000	− 260

δ^13^C remains constant in all scenarios, whilst AM was assigned as ^14^C-carbon dead in the first scenario and the oldest Δ^14^C obtained in this study for the second scenario.

**Figure 4:**
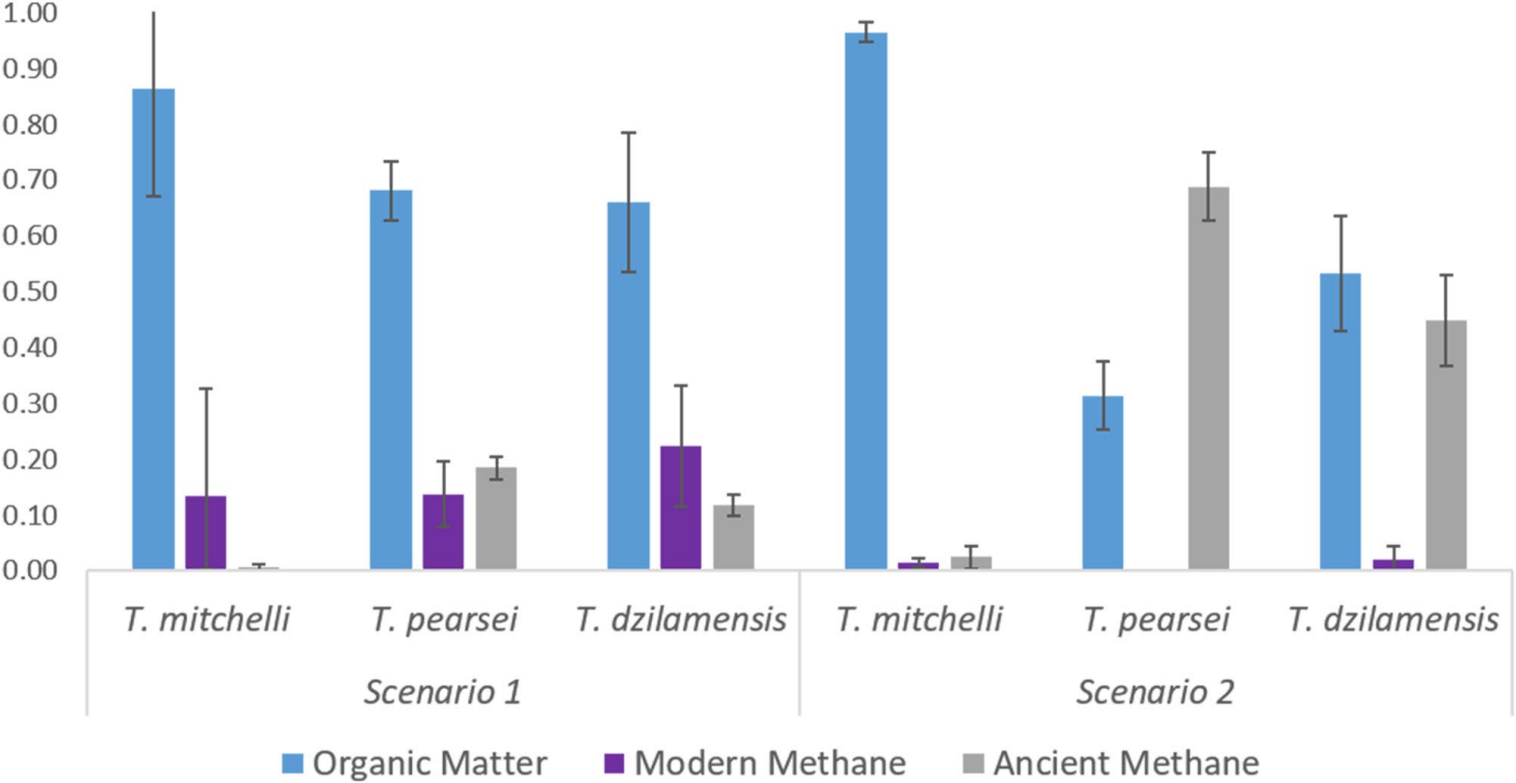
Relative contribution to the carbon in *Typhlatya* species as an average percentage of their carbon uptake in two mixing model scenarios, considering the Δ^14^C and δ^13^C of three potential feeding sources: organic matter (OM), modern methane (MM) and ancient methane (AM). Scenario 1 considered AM as ^14^C-dead carbon (Δ^14^C_AM2_ =  *− *1,000‰); scenario 2 considered AM as Δ^14^C =  *− *260‰ corresponding to the DIC measured in the saline layer of Ponderosa cave.

Under the first scenario (Fig. 4), the OM was the greatest carbon source for all three species: 88–98% for *T. mitchelli*, 66–74% for *T. pearsei* and 53–79% for *T. dzilamensis*. Modern methane (MM) contributed 1–11% of carbon to *T. mitchelli*, 7–18% to *T. pearsei* and 12–34% in *T. dzilamensis*. Ancient methane (AM) contributed with 0–1% of carbon to *T. mitchelli,* 16–20% in *T. pearsei* and 9–13% in *T. dzilamensis* (Fig. 4). The second scenario shows that OM in this scenario was again the greatest source of carbon for *T. mitchelli* (93–98%), but for *T. dzilamensis* (44–67%) and *T. pearsei* (26–38%) this source was greatly reduced compared to the first scenario. MM contributes marginally to the carbon sources in *T. mitchelli* and *T. dzilamensis* with 1–3% and 0–5% respectively, and shows no contribution to the biomass of *T. pearsei*. AM would contribute with 0–6% of carbon to *T. mitchelli*, as much as 62–74% to *T. pearsei* and 33–51% of carbon to *T. dzilamensis* (Fig. 4).

### Diel vertical distribution of *T. mitchelli*

**Figure 5:**
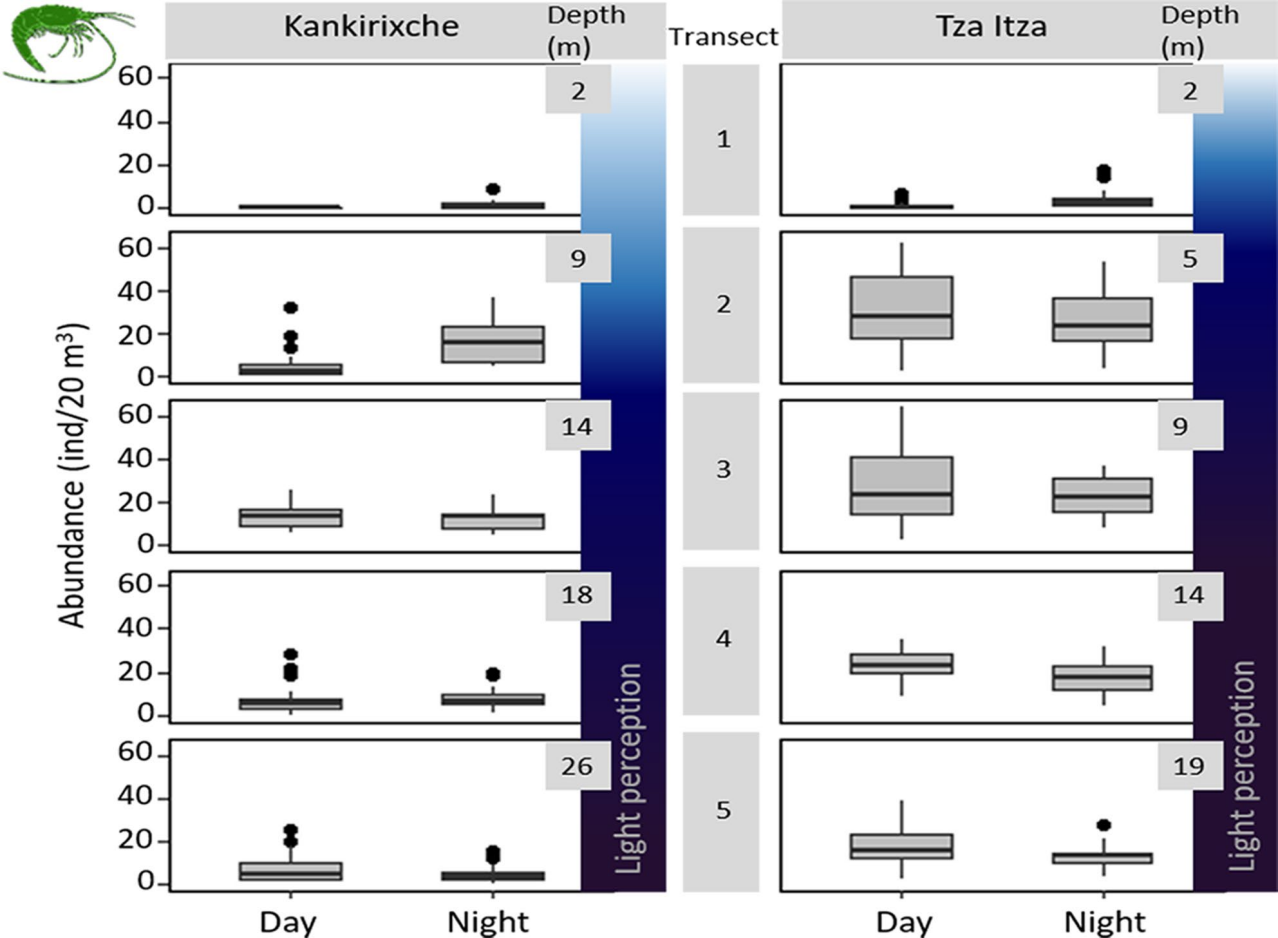
Distribution patterns of *T. mitchelli* through a depth gradient in day and night observations at Kankirixche and Tza Itza. Transect depth is shown in meters and relative to increasing transect number; transect 1 (under cenote pool) to 5 (end of cavern closest to cave). A light perception bar is drawn to represent the approximate depth at which light reaches in either cavern. At each transect abundance was registered during day and night in each system. The boxplots indicate the first and third quartile of the data, the black line shows the median and the whiskers extend to the most extreme data which is no further than 2 standard deviations, data beyond whiskers is shown as black dots. Artwork by Alberto Guerra.

The vertical distribution of *T. mitchelli* in the water column was determined by the presence of light and differed significantly between the two studied systems: Tza Itza and Kankirixche (F = 6.06; *p* < 0.001; Fig. 5). Overall, the frequency of occurrence of *T. mitchelli* in transects generally decreased as depth and distance from the cenote pool increased, with higher numbers closer to the cenote pool and lower numbers at the greatest depth of the caverns. A noteworthy exception, however, was the low occurrence of *T. mitchelli* in the shallowest transects (i.e. those immediately below the surface of the cenote pool) in both Tza Itza and Kankirixche.

Day/night differences in shrimp abundance were evident in shallow transects, where light was most intense, but were imperceptible in deep cavern transects where light was almost absent throughout a 24 h period (Fig. 5). In Kankirixche, the first two transects were markedly affected by day-night cycles, whereas in Tza Itza these daily differences were only observed in the first transect of the cenote pool (Fig. 5). Individuals were rarely observed during day observations in shallow transects of both cenotes, but numbers increased during the night, clearly indicating a preference for dark environments (Fig. 5).

**Figure 6:**
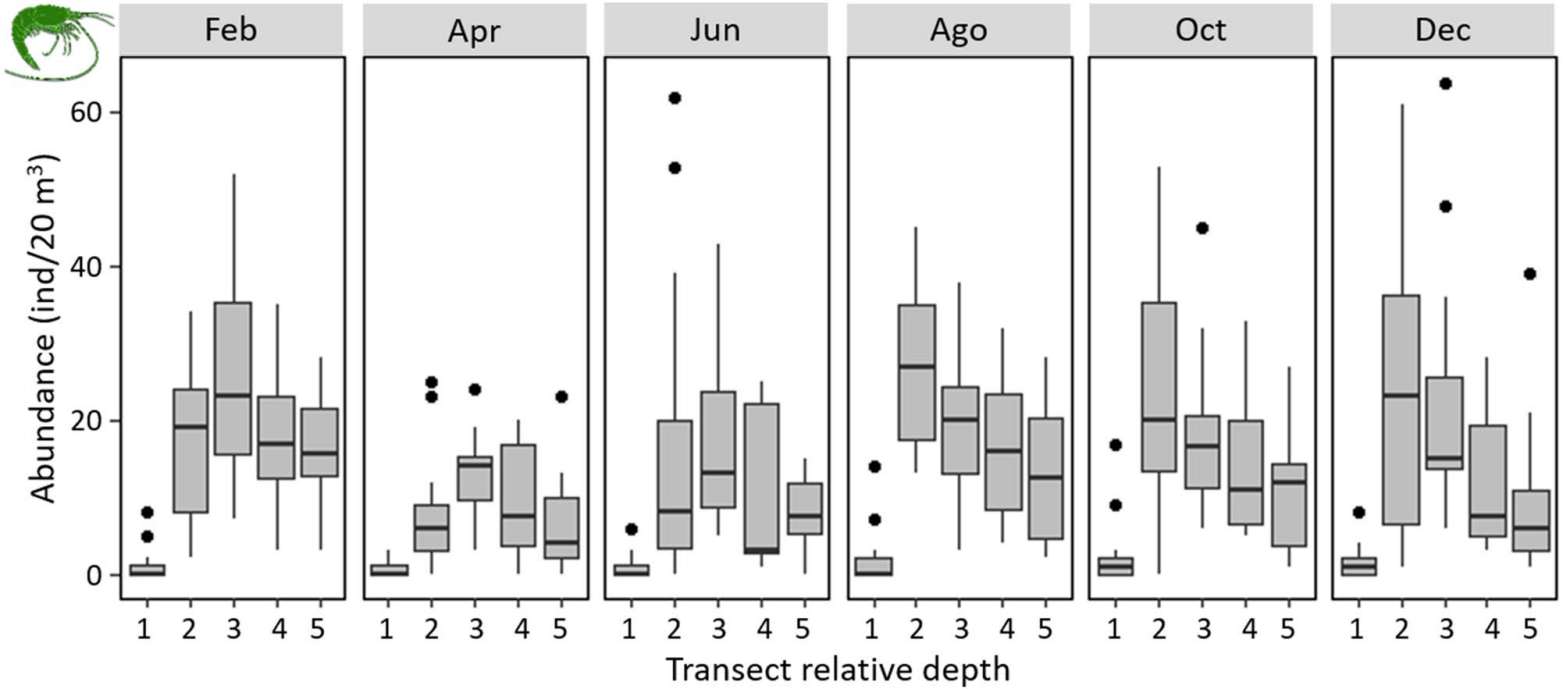
Depth distribution patterns of *T. mitchelli* throughout the year in Tza Itza and Kankirixche. Transect depth is shown as relative to each other and not as actual depth: transects 1 were at the cenote pool, transects 2–5 were distributed in an increasing depth gradient throughout the caverns of Tza Itza and Kankirixche systems. Boxplots indicate the first and third quartile of the data, the black line is the median and the whiskers extend to the most extreme data which is no further than 2 standard deviations, black dots represent counts which were beyond 2 standard deviations. Artwork by Alberto Guerra.

Both the total abundance and vertical distribution of *T. mitchelli* varied through the year (F = 5.71; *p* < 0.001) and did so in a similar fashion in Tza Itza and Kankirixche (F = 2.93; *p* = 0.087). Total abundance of *T. mitchelli* was highest during the months of August, October and February. These months correspond to the rainy season and the end of the winter season, where precipitation is highest in the Yucatan Peninsula. April (dry season), by contrast, had the lowest overall abundance (Fig. 6).

The vertical distribution from August to December showed a consistent pattern of high abundance in the cavern nearest to the cenote pool, decreasing as depth increased (Fig. 6). This pattern, however, started to flatten during February (end of winter) and was much less evident from April to June, when shrimp were more uniformly distributed throughout all but the shallowest transects (Fig. 6).

## Discussion

*Typhlatya* species are found throughout marine and fresh groundwater habitats and are some of the most abundant and widespread stygofauna component in the anchialine ecosystems of the Yucatan Peninsula^3,15,18–20^. In contrast with previous authors suggesting that anchialine fauna has no distribution patterns within underwater caves^21^, results herein show differently. Shrimp abundance varied markedly in space and the resulting patterns differed from one system to another depending on topographic features, solar influence and geographical position (Fig. 2B). In addition, we found both diel and seasonal variations in the vertical distribution and abundance of *T. mitchelli*, one of the most common species in the study area^3,15^. Furthermore, carbon source analysis show a distinct feeding pattern across *Typhlatya* species. Overall, our observations on three *Typhlatya* species in four groundwater systems of Yucatan portray a niche partitioning where salinity appears to play a fundamental role in separating the realized niche of *T. dzilamensis* from that of *T. mitchelli* and *T. pearsei*, whilst solar influence, food selection, space, and diel behavior would partitions the later species´ ecological niche. The upcoming discussion focuses on the biological processes that could explain the patterns identified in the present study.

One of the most outstanding characteristics of the karstic anchialine ecosystems in Yucatan is the vertical stratification of water masses, where the marine intrusion from the coast infiltrates under the meteoric water that infiltrates underground, resulting in clearly defined layers with marked salinity changes. Salinity gradients in aquatic habitats are considered one of the most important limiting factors of species distributions^22^. Changes in environmental salinity may impose severe physiological stress^23^. Therefore, salinity commonly governs geographical distributions, adaptive radiations, speciation and physiology^22,24^. Whilst crustaceans are known to have originated in the ocean, atyid shrimps have a long history since the colonization of freshwater environments and their systematics have revealed frequent cave invasions^4,25,26^. Studies on *Halocaridina rubra* from anchialine ponds in Hawaii, which are subject to marked daily fluctuations in environmental salinity, have shown these shrimp maintain constitutively activated mechanisms of ion regulation and high cellular osmoregulation in the gills regardless of salinity^24^. *Typhlatya*, as an anchialine cave restricted genus^25^^,^ must have developed a series of adaptations enabling their survival in coastal caves. Furthermore, speciation in Yucatan’s *Typhlatya* must have resulted in physiological, biochemical and genetic adaptations that derived in a different tolerance to salinity between closely related species and the colonization of subterranean habitats which are heterogeneous in depth and distance from the coast. Our results show that these shrimps are distributed according to salinity: *T. mitchelli* was found in all sites exclusively in the freshwater layer, *T. pearsei* was only observed in the fresh water at Nohmozon, and *T. dzilamensis* was only found in saline water at Ponderosa. The distribution patterns of *Typhlatya* species observed in this study constitute initial evidence in support of a physiological differentiation among species. Future research in adaptive physiology in response to salinity is key to reveal the osmoregulatory mechanisms and bioenergetics that will further explain the habitat selection (or limits) of these anchialine species (Chávez-Solís et al. *in prep*.).

It is noteworthy that *T. pearsei* was most abundant in the system with the highest dissolved oxygen in our study (Fig. 1). Perhaps, *T. pearsei* is more sensitive to hypoxia than the rest of its congeners. If this hypothesis is true, future studies should demonstrate a reduced metabolic capacity of *T. pearsei* under hypoxia, whereas *T. mitchelli* and *T. dzilamensis* should be comparatively less affected. Moreover, *T. mitchelli* should present a higher physiological performance, enabling to outcompete *T. pearsei* in hypoxic freshwater environments. Correspondingly *T. pearsei* should outcompete *T. mitchelli* under normoxia. Research on tolerance to hypoxia in stygobionts could test these predictions and provide a deeper understanding of the distribution patterns and adaptation mechanisms to dissolved oxygen variations in caves.

Despite the importance of oxygen and salinity determining the distribution patterns of many crustacean populations^22,27^, it does not explain the vertical and horizontal distribution of *Typhlatya* species in systems without a halocline. Abundance differences of *T. mitchelli* with depth in both Tza Itza and Kankirixche (Fig. 5) suggest that other explanatory variables are involved. Whilst light would appear to be a poor candidate explaining the distribution of blind stygobionts, negative phototaxis, as suggested by the results in the present study, has also been observed in anophthalmic stygobiont beetles *Paroster macrosturtensis* from Australian calcrete aquifers^28^. Evidence of this behavior is supported by observations of *T. pearsei* in the cenote pool at Nohmozon occurring only at night. In addition, transects nearest to the surface in Tza Itza and Kankirixche (i.e. those where sunlight had its greatest influence) consistently had low occurrence of *T. mitchelli*, particularly during day observations (Fig. 5). Furthermore, day/night differences in the abundance of *T. mitchelli* in Tza Itza and Nohmozon were limited to the shallow transects, were light influence was strongest. Differences in the way direct sunlight enters and reaches the water surface at Kankirixche compared to Tza Itza, together with the negative phototaxis, could account for the variations in the daily patterns of *T. mitchelli* observed between these two systems. Measuring traces of light in a cavern with commercial instruments can be challenging. Mejía-Ortiz et al.^29^ implemented an elegant solution by using long exposure photographs to show trace light at different depths in a dry cavern. Automated light quantification, however, is needed to determine whether light intensity triggers diel behavior in *Typhlatya*.

Diel migrations and nocturnal activity as that observed in this study have been previously reported in other blind stygobitic crustacean species, such as *Creaseria morleyi*^30,31^, *Halocaridina rubra*^32^, and *Hadenoecus subterraneous*^33^. Species restricted to caves are generally characterized by the reduction of visual structures and are part of the common troglomorphic features observed in stygobionts. Although some vestigial eye structures are observable in *Typhlatya*, no sign of visual function or pigments are evident, suggesting these species could be grouped as microphthalmic, or even anophthalmic (sensu Friedrich^33^). Whilst the assumption of anophthalmy—defined as “the lack of eyes at any stage of the life cycle and across populations”—is based on the absence of peripherally observable eyes, it may overlook vestiges of internalized visual organs and does not exclude the existence of other extra-retinal photoreceptors^33^. The negative phototactic behavior as a probable cause for the diel migrations described in the present study must find support in a mechanism of light detection amongst *Typhlatya* or closely related species. If these shrimps are still capable of perceiving light despite their visual reduction, then the way light reaches the water column will have a relevant role in keeping the circadian clock tuned, hence activity confined exclusively to dark hours night. Our observations also suggest that populations inhabiting the aphotic cave hydro-regions are present regardless of the time of day or night, as in *T. dzilamensis*. The constancy of biotic and abiotic parameters in this region may prevent the synchronization of the biological clocks of stygobionts^33^. The lack of a synchronizer in a cave population could result in a shift of their circadian rhythm producing an unsynchronized circadian rhythm among the population, an arrhythmic biological clock^33^^,^ or a reduction of sleep duration^34^. Our recurrent observations of *T. dzilamensis* in caves at any given time of day or night is consistent with any of these scenarios. Research on the anatomy of the eye, the nervous system, photoreceptors, biological clocks and genetic expression in *Typhlatya* is needed to further explain the differences in behavior and activity patterns observed both among and within these species.

A possible contributing factor to *Typhlatya* diel behavior in the cenote pools and light influenced caverns could be related to the presence of epigean and stygobitic predators that may also influence the distribution and size of prey populations. Predation has been shown to modify prey behavior by inducing vertical migration patterns or forcing prey to retrieve to refuges during light periods^32,35^. Predators in cenote pools include a diverse array of freshwater fish^36^ and other stygobionts, such as *Ophisternon infernale*, *Typhlias pearsei*, *Creaseria morleyi* and the stygofile *Rhamdia guatemalensis*, all of which were recorded during night observations in the present study.

Habitat preference in the underground ecosystems is certainly linked to a number of ecological tradeoffs. A balancing component for blind prey living in the sun influenced hydro regions (thus an easy prey) could be the access to recently deposited plant debris rich in nutrients, or algae which are high in nitrogen content and easier to assimilate than plants^10,37^. This could be selecting *T. mitchelli* and *T. pearsei* to remain close to the cenote pools.

Advantages of shallow waters could also be a greater amount of dissolved oxygen and other organic inputs that are unlikely to reach the cave passages. If cenote pools are the only place in anchialine systems where photosynthesis takes place and constitute sinkholes for allochthonous input, then these hydro regions represent a nutrient attraction for cave primary consumers. Stygobionts in this trophic level would increase in density at cenote pools, further attracting epigean and hypogean predators. Results in this direction would suggest that cenote pools are “feeding hotspots” for all species in the heterotrophic anchialine ecosystem. If, on the other hand, photosynthetic and allochthonous nutrient input is scarce or absent in the cenote pool or represents a decimating risk due to visual predators, then *Typhlatya* must find food in the oligotrophic caves. If the aphotic dwelling *T. dzilamensis* deep inside caves has developed a strategy to incorporate in situ production sources (such as chemosynthesis or methane derived biomass) to their diet, then most individuals would keep away from the busy photosynthetic *hot-spots*.

Caves are considered oligotrophic because of a severe and almost constant scarcity of food. Additionally, bacterial mats have been suggested to yield lower energy transfer than that of photosynthesis^38^. Even so, a trade-off in energy transfer versus the risk of predation can be recognized. In either scenario, the anchialine ecosystems would appear to have a bottom-up control trophic structure, where the availability of autotrophs governs the abundance and distribution of the community. Our results show a greater abundance in hydro-regions linked to the surface (namely cenote pools and caverns), while cave populations were the least abundant throughout this study. Stable isotopes and radiocarbon analysis would link the distribution patterns herein observed with the available feeding sources and the importance of these sources to each of the species.

Metabolic pathways in autotrophs have different isotopic fractionation rates—a differential uptake of isotopes—which create specific carbon-isotope fingerprints^11^. δ^13^C values of consumers reflect those of their feeding sources and will be passed on to higher trophic levels, enabling the reconstruction of food webs^9–11^. The wide range of δ^13^C and Δ^14^C values observed in *Typhlatya* species collected from fresh groundwater (FGW) and saline groundwater (SGW) environments suggests a mixed contribution of photosynthetic and chemosynthetic derived matter, as well as modern and ancient carbon contributing to their biomass. Nevertheless, our results show each species has a specific carbon composition indicating a differential food proportion from each of the available sources.

The range of C/N ratios of < 10 indicates that these species primarily obtain their carbon from algae derived organic matter (OM), rather than terrestrial plant derived OM^39^. To our knowledge, this ratio has not been evaluated for bacterial mats or methane oxidizing bacteria. C/N ratios indicate *Typhlatya* feeds mainly on algae or aquatic derived matter, but to further understand the differences and precise nature of their feeding sources, stable isotopes and radiocarbon analysis were implemented.

Bulk δ^13^C in *T. mitchelli* distributed in the cavern show values similar to those of photosynthetically derived OM (i.e. algae, phytoplankton and C3 plants). The old apparent age of dissolved inorganic carbon (DIC) in fresh groundwater (FGW) in Tza Itza, contrasts with the modern carbon age of *T. mitchelli* collected from this system. This suggests that the carbon source of *T. mitchelli* in Tza Itza is not using ancient DIC from the dissolution of the limestone, but rather a modern source of carbon, most likely obtained through the assimilation of modern CO_2_ by freshwater algae. Precipitation and gas diffusion at the water surface could fuel the algae and phytoplankton production with modern CO_2_ and result in a modern-aged carbon assimilation by the *T. mitchelli* which fed on such sources. Furthermore, the mixing model based on stable isotopes and radiocarbon suggests that *T. mitchelli* incorporates the vast majority of its carbon from modern OM (Fig. 4).

Seasonal variations in the abundance and vertical distribution of *T. mitchelli* in Tza Itza and Kankirixche (Fig. 6) are in accordance with the rainy seasons, suggesting a strong dependence on external OM inputs. Overall, the vertical distribution of *T. mitchelli*, decreases in depth as probably allochthonous input of OM is not carried into the deepest region of the system. Modern OM is a carbon source that is most available during the rainy season, when modern CO_2_ and allochthonous sediment inputs increase in the cenote pools. Seasonal shifts of allochthonous inputs have been suggested to modify dietary selection in stygobiont beetles which showed a marked difference between dry and rainy seasons^40^. Considering that more than 90% of *T. mitchelli* diet derives from a photosynthetic origin, a reduction in allochthonous input or photosynthetic in situ production could represent a reduction of food availability, resulting in the decline of its population size or a shift in its distribution, as observed in Kankirixche and Tza Itza, where the population was smallest and mainly distributed at deeper transects during dry months (Fig. 6). These hypotheses are in accordance with our stable- and radiocarbon isotope analyses, which show this species is predominantly feeding from modern and photosynthetically derived sources associated to the surface and, their distribution is predominantly higher in the caverns, closest to the cenote pools.

Bulk δ^13^C of *T. dzilamensis* (collected from the cave in saline groundwater of Ponderosa) and that of *T. pearsei* (collected in FGW from the cenote pool of Nohmozon), reflect extremely negative values which cannot be derived alone from pathways such as photosynthesis, ammonium or sulfur oxidation, and thus, are most likely derived from methane oxidization^41^. Furthermore, chemoautotrophic bacteria from caves in Yucatan and Quintana Roo have been reported to have a δ^13^C trace ranging from − 25 to − 46‰^8,10^, which is in accordance with our δ^13^C results from *T. pearsei* and *T. dzilamensis*. Observing a similar δ^13^C trace in both *T. pearsei* and *T. dzilamensis* suggests they are incorporating a portion of their carbon from a non-photosynthetic microbial source that is present both in the exclusively freshwater system of Nohmozon and in the anchialine system of Ponderosa (Fig. 3). Our δ^13^C results indicate both *T. pearsei* and *T. dzilamensis* are not exclusively feeding from methane derived sources, but rather a combination of the lighter source, such as methane (δ^13^C ≈ − 66‰) and a heavier photosynthetic derived carbon source, such as algae or plant derived.

The combined determination of ^13^C and ^14^C from *Typhlatya* tissue reflects that each species obtains carbon from a different source or from the same source but in different proportions. Adding the use of radiocarbon analysis in each species allowed the identification of the modern and ancient carbon sources from which they are feeding. The first of our mixing model scenarios suggests that all *Typhlatya* species preferentially consume carbon derived from organic matter (OM). The isotopic composition in *T. mitchelli* is intimately related with a modern derived photosynthetic source (C3 plants or algae) and only a marginal proportion is assimilated from other sources (Fig. 4). *Typhlatya pearsei* and *T. dzilamensis*, under this model, incorporate greater proportions of MM and AM in addition to OM, as compared to *T. mitchelli*. In addition to OM and MM, there is a partial contribution to the biomass of all *Typhlatya* from AM, derived most likely from ^14^C-dead carbon. It is noteworthy that *T. pearsei* and *T. dzilamensis* show similar carbon source incorporation of both δ^13^C and Δ^14^C, despite the fact they inhabit systems with different salinity and are several hundred kilometers apart. These results are in accordance with the hypothesis that biogeochemical processes in groundwater ecosystems are likely to be universal, as observed in other systems around the world^8,42–44^.

Our second mixing model scenario considers an alternative carbon source for the ancient methane provided by the bacterial reduction of the available DIC. This is an alternative source of carbon in anchialine systems, such as Ponderosa. The carbonate reduction pathway may not be important for *T. mitchelli* in FGW environments, since the DIC isotopic values of the freshwater in Tza Itza are strikingly different to the bulk biomass isotopic readings. Nevertheless, this pathway would increase its importance in fresh groundwater if other substrates were limited^41^, as suggested by the isotopic readings of *T. pearsei* in Nohmozon. The carbonate reduction pathway, by contrast, should be dominant in saline environments, where organic carbon is scarce^41^. This second scenario shows how important DIC incorporation can be to *T. pearsei* and *T. dzilamensis* given that the incorporation of AM derived carbon markedly increases as OM incorporation decreases when compared to the first scenario. This reduction could suggest a reduced availability of OM in the cenote pool of Nohmozon and in the saline layer of the Ponderosa cave hydro region, driving *T. pearsei* and *T. dzilamensis* into feeding from alternative sources.

Given that the analyzed *T. pearsei* were collected from the cenote pool of a FGW system, it seems counterintuitive that it would be feeding on similar sources than *T. dzilamensis* which inhabit the saline region under the halocline. A higher pH, as observed in Nohmozon, facilitates the production of carbonates and reduces the dissolution of CO_2_, possibly contributing to a greater availability for older DIC for primary producers to assimilate. Nevertheless, further research on the available sources is necessary to determine the genetic identity of these chemosynthetic or photosynthetic ancient-carbon feeding sources.

Combined results from the mixing models show *Typhlatya* species are feeding from shared sources in different proportions, thus identifying a niche partitioning among these closely related congeners. In freshwater environments, *T. mitchelli* feeds almost exclusively on photosynthetic derived sources while *T. pearsei*, under the first scenario, complements its diet with roughly a third of methane derived matter (MM and AM). In the saline groundwater *T. dzilamensis* has a similar dietary proportion uptake as *T. pearsei*, with approximately a third of its diet coming from methane derived sources. Identifying their feeding sources helps to explain the observed specific distribution patterns within the anchialine systems and accounts for the widespread observation of *Typhlatya* throughout the Yucatan underground.

A different selection of food sources by *Typhlatya* species indicates an ecological niche separation. It has been suggested that the degree of trophic specialization is expected to decrease in low productivity environments resulting in omnivorous or generalist species^45^. Our results are in accordance with these assumptions as *T. mitchelli*, which is distributed in the vicinity of sinkholes and would most likely have the greatest feeding diversity, is feeding almost exclusively on OM input. On the contrary, *T. dzilamensis* distributed in the aphotic passages which have the least OM input, compliments its diet with modern and ancient methane derived sources (Fig. 4). *Typhlatya pearsei* in Nohmozon is an extraordinary case, as it would be expected to have abundant availability of OM input and production, yet it is also complimenting its diet with modern and ancient methane derived carbon. The broader range in feeding sources of *T. pearsei* could be linked to a competition strategy that enables it to coexist with *T. mitchelli* in freshwater environments.

Such apparent differentiation on food sources must of course be accompanied by potential differences in foraging and feeding behavior. Although these are difficult to record and observe in situ, it could also be that selective forces related to the access to slightly different food sources has selected for different morphologies in mouth parts, which are much simpler to observe and compare. With the findings of the present work we hypothesize that a quantitative comparison of morphological variables associated with the filtering and scraping of bacterial mats and biofilms will discover significant specialization on the setae and dactyls between these 3 species.

## Closing remarks

What we observe today in these anchialine caves is, indeed, the result of co-evolution of these species with the geologic history, environmental change and biologic interactions within these underground habitats. Overall, combined results of species distribution, stable isotopes and radiocarbon analysis, and diel behavior obtained in this study have shown how *Typhlatya* species in Yucatan have partitioned their niche to coexist throughout evolution. Niche partition between these sister species shows their specific role and importance in energy transfer to higher trophic levels and highlights the exclusion distribution patterns within groundwater ecosystems. These results help understand the wide distribution of *Typhlatya* species throughout the Yucatan Peninsula. The salinity preference of *Typhlatya* species, nevertheless, impels further research in the physiology of *Typhlatya* and raise questions as to whether the halocline is such a barrier that would drive allopatric speciation. It now becomes crucial to understand the physiological traits and adaptations that separate these species into different habitats and ecological niches.

## Methods

Surveys were performed in Tza Itza (20.730311° N, 89.46608° W), Nohmozon (20.623266° N, 89.384207° W), Ponderosa (entering from cenote Xtabay 20.499183° N, 87.260842° W) and Kankirixche (20.637306° N, 89.632892° W). Selection of these four systems was based on their contrasting hydrological, morphological and biological features that make each of them singular, thus, systems are not considered random replicates of each other. These systems represent two distinct hydrographic basins. Ponderosa lies in the Quintana Roo basin, separated from the rest of the Yucatan Peninsula by the Holbox fracture, whereas, Tza Itza, Nohmozon and Kankirixche are located in the southern section of the ring of cenotes in the Yucatan basin^46^. Vertical profiles of salinity, temperature, pH, redox potential and dissolved oxygen in Tza Itza, Nohmozon and Ponderosa were obtained using a multiprobe Hydrolab DS5X. Because diving may itself influence environmental conditions in caves^47^, only measurements recorded by the multiprobe in its way into the cave were considered, whereas those taken returning towards the surface were discarded. Previous studies revealed that these physical and chemical properties are markedly constant through time^48^, hence measures were only recorded once during our survey. Trials using Hobo data loggers were unable to measure light in the cavern even when it was noticeable by the naked eye. Consequently, light was recorded by observers as present or absent where transects were installed.

**Figure 7:**
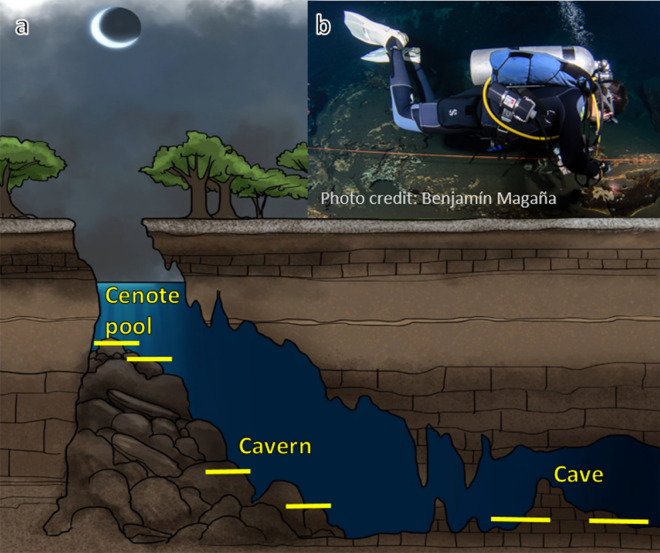
(**A**) Conceptual model of groundwater hydro regions depicting the general profiles of transects used to monitor *Typhlatya* abundance and distribution within the systems: cenote pool, cavern and cave. Two transects (yellow lines) were installed in each hydro-region and traversed simultaneously by two divers, twice during each of three seasons (dry, rainy and winter) throughout 1 year. (**B**) Diver approaching a transect in the cavern of Kankirixche. Artwork by Alberto Guerra.

In order to identify patterns in the distribution of *Typhlatya*, we divided each system into three “hydro-regions” considering light penetration and the external influence of runoff water and organic matter from the surroundings. The *cenote pool* was defined as the region under the water surface that receives direct influence from the surroundings i.e. the air interface, organic debris, interaction with terrestrial animals and, in some cases, direct or indirect sun light. The *cavern* was defined as the transition zone from the cenote pool to the cave: a region where sunlight is still perceivable but has no direct incidence, there is less external influence and has no vertical access to the surface. The *cave* was defined as the aphotic region, with minimum or no influence from external factors and no vertical access to the surface (Fig. 7).

### Spatial distribution of *Typhlatya* species throughout the anchialine system

To characterize the distribution of *T. mitchelli, T. pearsei* and *T. dzilamensis* at Tza Itza, Nohmozon and Ponderosa, two underwater transects (sensu Sutherland^49^) in each of the three hydro-regions (cenote pool, cavern and cave) were used (Fig. 7A). Each transect was temporarily deployed installing a 10 m rope close to the ground and traversed simultaneously by two divers, one on each side at one meter above the ground. The number of individuals of *Typhlatya* species encountered as far as 2 m away from each side of the rope were registered. Only those individuals that were unequivocally identifiable underwater as pertaining to any of the species examined were considered. This resulted in 20 m^3^ surveyed by each diver in each transect. Sampling procedures were repeated twice in each of the three meteorological seasons previously described for limnologic systems in the Yucatan Peninsula^48^: warm–dry (March–May), winter-storms with occasional short showers (November–February) and rainy seasons (June–October). All observations were performed after dark.

A multivariate approach was used to describe changes in *Typhlatya* species composition through the systems, hydro-regions and seasons examined. A non-metric multi-dimensional scaling (nMDS) procedure was used to order (n = 402) samples in a 2-dimensional configuration. The Bray–Curtis similarity index was used to obtain the resemblance matrix of previously log-transformed count data (log (x + 1)). Differences in species composition were then analyzed using a multivariate analysis of variance (followed by pairwise t-test when relevant) with the following underlying factorial model: System, fixed factor with 3 levels (Tza Itza, Nohmozon and Ponderosa); Season, fixed factor with 3 levels (dry, rainy and winter); and Hydro-region, fixed factor with 3 levels (cenote pool, cavern and cave). The observer was taken as a random factor (with 2 levels) nested within each three-way combination of system, hydro-region and season. A total of 9,999 permutations of residuals under the reduced model were used to obtain empirical distributions of F values. Statistical analyses were performed using PRIMER software and PERMANOVA add in^50^.

### Carbon tracing in *Typhlatya* tissue

In order to study the potential contribution and relative age of different carbon sources to the biomass of *Typhlatya* species, accelerator mass spectrometry (AMS) δ^13^C and Δ^14^C from individuals of *T. mitchelli*, *T. pearsei* and *T. dzilamensis* (obtained respectively from Tza Itza, Nohmozon and Ponderosa, under collection permit SEMARNAT/SGPA/DGVS/004471/18) were analyzed in the Accelerator Mass Spectrometry Laboratory (LEMA) at the Instituto de Física of the Universidad Nacional Autónoma de Mexico (UNAM). δ^13^C and Δ^14^C from dissolved inorganic carbon (DIC)—carbon dioxide (CO_2_) and carbonates (CO_3_)—were measured in additional samples of fresh groundwater (FGW) from Tza Itza, Nohmozon and Ponderosa, and saline groundwater (SGW) from Ponderosa. Values were then related to carbon isotopic ratios from known sources that have been measured in cenotes and caves in Yucatan, and which could be potential feeding sources for *Typhlatya*.

The *Typhlatya* samples were cleaned with ultrapure water, treated with HCl 0.5 M to remove inorganic carbon, salts and other adhered contaminants, rinsed again with ultrapure water and then freeze-dried. For ^13^C and ^14^C analyses, samples were processed in an automated graphitization equipment (AGE III from Ion Plus) where the carbon was first converted to CO_2_ and then to pure graphite. Carbon and Nitrogen content was estimated in the AE (Elementar vario MICRO cube) from the AGE III, as weight percent of the total sample mass. Oxalic acid II (NIST SRM 4990C) was used as a primary standard and Phthalic acid (with no ^14^C) was used as a blank. Analysis of ^14^C, ^13^C and ^12^C of the obtained graphite was performed in a 1 MV Accelerator Mass Spectrometry (AMS) High Voltage Europe Engineering (HVEE) at LEMA^51^.

The content of ^13^C, reported as δ^13^C is the difference of a sample ^13^C/^12^C relative to Vienna Pee Dee Belemnite (PDB) carbonate standard and is expressed in parts per thousand (‰)^52^.

$$\updelta ^{13} {\text{C}} = (^{13} {\text{C/}}^{12} {\text{C}}_{{{\text{sample}}}} -^{13} {\text{C/}}^{12} {\text{C}}_{{{\text{standard}}}} {)/}^{13} {\text{C/}}^{12} {\text{C}}_{{{\text{standard}}}} \times 1000$$

The content of ^14^C is reported as ∆^14^C; the deviation of a sample ^14^C/^12^C ratio from its atmospheric preindustrial level expressed in parts per thousand (‰)^13,53^, and the precision of ^14^C measurements was ± 3‰ for modern sample. Measured ^14^C/^12^C isotopic ratios were corrected for isotopic fractionation using the ^13^C/^12^C isotopic ratios measured in the accelerator. Corresponding radiocarbon contents (apparent age) were calculated using computer codes developed at LEMA^51^. When measuring δ^13^C by AMS, some isotopic fractionation can be introduced through the sample preparation and AMS measurement. Therefore, a subsample of *Typhlatya* individuals was halved to compare δ^13^C by AMS and by isotope-ratio mass spectrometry (IRMS) which is the standard technique for δ^13^C analysis. Two replicates of δ^13^C IRMS analyses were performed in a Delta V Plus Thermo Scientific equipment at the Laboratorio de Análisis de Isotópos Estables at the Unidad Académica de Ciencias y Tecnología in Yucatan of the UNAM.

#### Mixing models

In order to explain the range of δ^13^C and ∆^14^C values found in *Typhlatya* from Yucatan and considering that carbon fixation through methanotrophy has been suggested in similar sites^8,16^, we built a mixing model considering three potential carbon sources that could contribute to the food of *Typhlatya* species:

- Organic matter (OM), represents a modern carbon source that is fixed through photosynthesis by both aquatic algae and phytoplankton located in the cenote pool or by terrestrial plants in the immediate surroundings.
- Modern methanogenic carbon (MM) represents terrestrial methane produced by fermentation of methylated substrates.
- Ancient methanogenic carbon (AM) results in ^14^C depleted sources due to either the methanogenic decomposition of old organic matter by methane oxidizing bacteria (MOB), or the assimilation of ancient dissolved inorganic carbon (DIC) made available through the dissolution of limestone.

To assess the contribution of these carbon sources to *Typhlatya* species, biomass values of δ^13^C and ∆^14^C, as well as values published for potential sources were incorporated into the IsoSource mixing model software^54^. We considered two possible scenarios in which the supply of ancient methane could be fueled by either (1) organic ^14^C-dead carbon, or (2) the oldest ^14^C measured from DIC in monitored systems. Values of δ^13^C were fixed for both scenarios: δ^13^C_OM_ represents a mix of photosynthetic organic matter from terrestrial and aquatic sources, δ^13^C_MM_ corresponds to a fresh water bacterial modern methane measured in anchialine caves^8^, and δ^13^C_AM_ corresponds to bacterial methane produced by carbonate reduction in a saline environment (taken from measurements in Quintana Roo caves^8^) (Table 3). OM was assigned the average Δ^14^C from the most modern biomass measured in this study, which reflects the modern signature of the terrestrial organic matter in this freshwater layer. Carbon derived from modern methane was considered as Δ^14^C_MM_ = 0‰. For the first scenario, a fossil value of Δ^14^C_AM1_ =  − 1,000‰ was assigned for ancient methane derived carbon, representing a ^14^C depleted source which would be older than 50,000 yBP (Table 3). The second scenario, maintained above described values except for the ancient methane derived carbon which was established as Δ^14^C_AM2_ =  − 260‰ corresponding to the average DIC measured in the saline layer of Ponderosa cave (Table 3).

### Diel vertical distribution of *T. mitchelli*

To explore possible variations due to daily and seasonal behavior-related migrations, we used *T. mitchelli* as a biological model in two systems that had a particularly high abundance and depicted different light regimes: Tza Itza and Kankirixche. Tza Itza is a cave-type cenote with a horizontal entrance and dry cave access, in which light only reaches the water surface indirectly. Kankirixche is a jug-shaped cenote with an opening directly above the water surface, through which direct sunlight reaches the water surface during part of the day.

**Figure 8:**
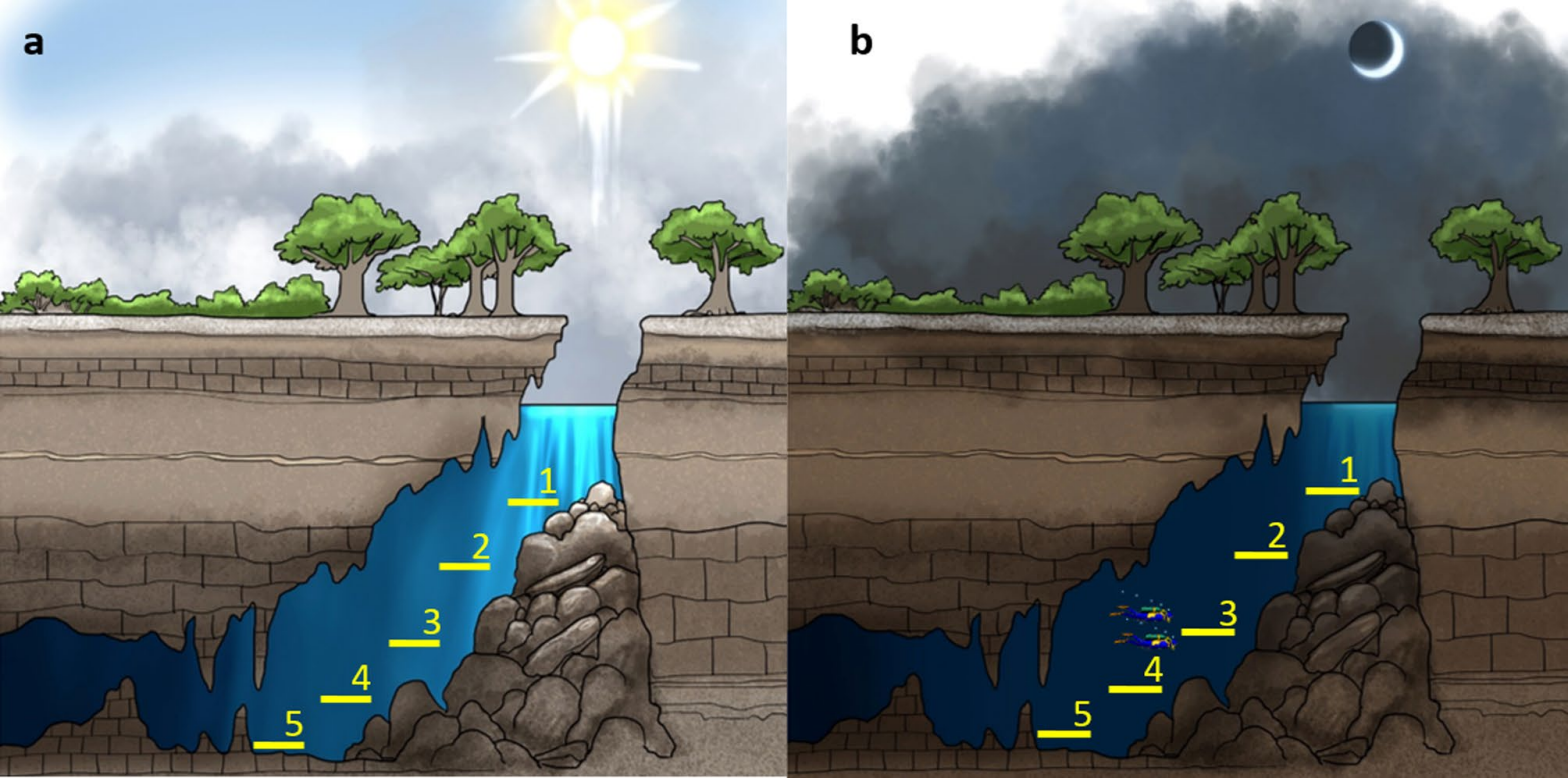
Diagram showing the location of sampling transects installed in Kankirixché and Tza Itza where the number of *Typhlatya mitchelli* were counted during day (**A**) and night (**B**) observations performed by two divers every 2 months throughout 1 year. Artwork by Alberto Guerra.

The number of *T. mitchelli* individuals was recorded using five transects which were located one at the cenote pool directly under the water surface, followed by four transects distributed in a depth gradient throughout the cavern (Fig. 8). Due to morphological differences between sampling sites, the deepest transect reached 19 m and 27 m at Tza Itza and Kankirixche, respectively. Each transect was monitored by two divers traversing simultaneously as described earlier. Sampling took place at approximately midday and 30 min after sunset, and was repeated twice every 2 months throughout a year. Differences in *Typhlatya* counts related to between-observer variation were first examined by introducing a random factor (observer) with two levels within each transect and testing its significance.

Changes in *T. mitchelli* distribution with depth were statistically analyzed using generalized linear models (GLM). The underlying experimental design was a three-factor model with “depth” (5 levels), “light” at the time of observation (2 levels: day and night), “system” (2 levels: Tza Itza and Kankirixche) and the “month” of the year (6 levels: February, April, June, August, October and December). Models were adjusted using a *Poisson* distribution with a log link function, and letting the over-dispersion parameter (rho) to be estimated by the model (i.e. using the “glm” R function with “family = quasipoisson”). The model with the best set of explanatory variables was obtained using a backward step procedure starting with the full model and testing the inclusion of each variable one by one^55^.

## Supplementary information

Supplementary information.
