# Supplementary material for: Distribution patterns, carbon sources and niche partitioning in cave shrimps (Atyidae: Typhlatya)
Source: Sci Rep. 2020 Jul 30;10:12812. doi: 10.1038/s41598-020-69562-2 (PMC7393362; doi:10.1038/s41598-020-69562-2)

# Supplementary material

**ST1.** δ^13^C results of halved *Typhlatya* samples analyzed by AMS and IRMS.

| **Sample code** | **AMS δ^13^C (‰)** | **IRMS δ^13^C (‰)** |
| --- | --- | --- |
| LEMA1459 | -23.0 ± 2.0 | -25.0 ± 0.2 |
| LEMA 1460 | -25.5 ± 1.1 | -25.7 ± 0.2 |
| LEMA 1461 | -25.9 ± 0.8 | -26.3 ± 0.2 |
| LEMA 1462 | -26.0 ± 2.0 | -26.2 ± 0.2 |

**SF1** Carbon and nitrogen composition *in tissue of* Typhlatya *species*. Centroids are shown as *shrimp* objects and represent the mean value.


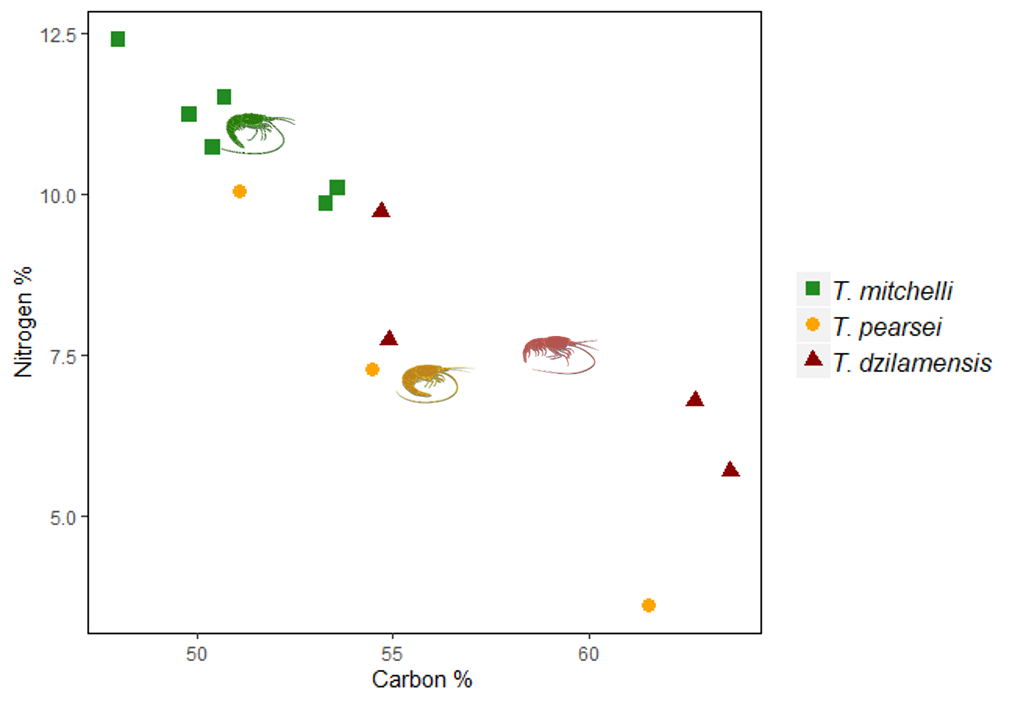

Supplement: Supplementary file 1 — Supplementary information. [file 41598_2020_69562_MOESM1_ESM.docx]
